# Respiratory syncytial virus-associated hospitalizations among children: an Italian retrospective observational study

**DOI:** 10.1186/s13052-024-01617-w

**Published:** 2024-03-07

**Authors:** Francesca Fortunato, Angelo Campanozzi, Gianfranco Maffei, Fabio Arena, Valeria Delli Carri, Tiziana Rollo, Pier Luigi Lopalco, Domenico Martinelli

**Affiliations:** 1 Hygiene Unit, Department of Medical and Surgical Sciences, Policlinico Foggia Hospital, University of Foggia, Foggia, Italy; 2 Pediatrics Unit, Department of Medical and Surgical Sciences, University of Foggia, Foggia, Italy; 3 Neonatology and Intensive Care Unit, Policlinico Foggia Hospital, Foggia, Italy; 4 Microbiology and Virology Unit, Department of Clinical and Experimental Medicine, University of Foggia, Foggia, Italy; 5 Microbiology and Virology Unit, Policlinico Foggia Hospital, University of Foggia, Foggia, Italy; 6 Department of Biological and Environmental Sciences and Technology, University of Salento, Lecce, Italy; 7 Department of Medical and Surgical Sciences, Department of Hygiene, University of Foggia, Policlinico Riuniti University Hospital of Foggia, Ospedale “Colonnello D’Avanzo” Viale degli Aviatori, 2, 71122 Foggia, Italy

**Keywords:** Respiratory syncytial virus, RSV, Epidemiology, Hospitalization, Bronchiolitis, Seasonal trend, RSV-A, RSV-B, Economic burden

## Abstract

**Background:**

Respiratory syncytial virus (RSV), a single-stranded RNA virus, is a leading cause of hospitalization in infants, especially ≤ 2 months of life. In the light new immunization strategies adoption, we described epidemiological and clinical characteristics of RSV-associated hospitalizations in pediatric and neonatal intensive care units of the Policlinico Foggia Hospital, Apulia Region, Italy.

**Methods:**

Hospitalized children with a laboratory-confirmed RSV infection from 2011 to 2023 were retrospectively evaluated. Clinical information was collected from Hospital Discharge Registry in the period 2011–2020. The proportion of the hospitalization for acute respiratory infections (ARIs) associated to RSV was calculated and the hospitalization cost was analyzed by using the diagnosis-related group reimbursement rate. The anticipated impact of immunization either with monoclonal antibodies or maternal immunization on the number of hospitalizations was estimated. All analyses and quality assessment were performed using STATA/SE15.0.

**Results:**

A total of 1,005 RSV-cases were included in the study, of which 86.3% occurred between December-March. In the period 2011–2020, 832 RSV-cases were matched with the corresponding hospital admissions; 75.2% were aged < 1 year (49.6% 0–2 months). Bronchiolitis was the most frequent admission diagnosis occurring in 63.3% of patients; 25% of children were affected by a very severe RSV-disease. Younger age ≤ 2 months (OR:14.8, 95%CI:8.30–26.31, *p* = 0.000), higher length-of-hospital-stay (OR:1.01, 95%CI:1.0–1.02, *p* = 0.030) and history of prematurity (OR:4.4, 95%CI:1.57–12.11, *p* = 0.005) were associated with a higher disease severity. RSV caused 48.9% of ARIs among children < 1 year. The mean cost of an RSV-associated hospitalization was 3,036 euros/year, with the higher cost in the 0–2 months age group (4,225 euros/year). Immunization programs with nirsevimab could prevent 51.4 RSV hospitalizations/year and 18.1 very severe RSV disease/year in infants < 1 year of age. RSV vaccine could prevent 46.1 of hospitalizations/year caused by RSV within 180 days after birth.

**Conclusions:**

Our study contributes to outlining the baseline profile of RSV-associated hospitalizations among Italian children by providing epidemiological/clinical/economic estimates. While awaiting new recommendations on immunization, healthcare-workers should persist in implementing public health measures and appropriate case management to control RSV seasonal epidemics. Strengthened laboratory RSV surveillance is needed to inform the implementation of the new immunization strategies.

**Supplementary Information:**

The online version contains supplementary material available at 10.1186/s13052-024-01617-w.

## Background

Respiratory syncytial virus (RSV) is a single-stranded RNA virus belonging to the Paramyxoviridae family. There are two major antigenic subtypes of human RSV (A and B), primarily determined by antigenic drift and duplications in RSV-G sequences [1]. RSV is a leading cause of lower respiratory tract infections and represents a major cause of morbidity and mortality worldwide in infants and young children under the age of five years [2]. The first infection may cause severe bronchiolitis that can sometimes be fatal [1]. Most infants are infected during the first year of life and nearly all of them by the age of two [3]. Premature infants and children with chronic diseases are at greater risk of RSV-associated severe illness. However, the majority of RSV-related hospitalizations are in healthy, term infants [3, 4].

A global review on RSV burden estimated 33 million RSV-associated lower respiratory infection episodes in 2019 (i.e., 3.6 million hospital admissions, 26,300 in-hospital deaths, and 101,400 RSV-attributable overall deaths) in children younger than 5 years [2]. In Europe, an average of around 250,000 respiratory hospitalizations were estimated in children under 5 years of age between 2006 and 2018, with 75% of cases occurring among children younger than 1 year [5]. According to the Italian surveillance network RespiVirNet, RSV caused 49.1% and 22.3% of influenza-like-illness in children during the 2022–2023 flu season in the < 2 and 2–4 years age groups, respectively [6]. Italian virologic surveillance for the 2023–2024 season identified a total of 2,218 RSV-positive samples in the first 11 weeks of collection, with the majority of cases in patients aged 0–2 years [7]. Additionally, Italian epidemiological studies confirmed a higher RSV incidence and higher need for neonatal intensive care unit (NICU) for a younger age (≤ 3 months) [8, 9].

RSV is a seasonal virus, whose epidemiology strongly depends on the climate zone. It generally circulates from October through early May in temperate regions of the Northern Hemisphere with a typical peak between December and February [5–10], as also showed by Italian epidemiological studies where the majority of cases occurred in these months [9, 10]. However, during the Sars-CoV-2 pandemic, the prolonged lack of viral exposure, decrease of social contacts but also potential SARS-CoV-2-induced immune dysregulation and viral interactions between SARS-CoV-2 and RSV have sharply changed this seasonal trend of RSV infections in children [11]. A marked decrease in RSV case detection were experienced in 2020–2021 season compared to previous winter seasons, followed by larger and/or earlier peaks in transmission in 2021–2022 [12].

Despite the significant burden associated with RSV disease, the only prevention strategy currently available is Palivizumab, a monoclonal antibody approved in 1999. Pavilizumab is available for immunoprophylaxis in a limited high-risk infant population, leaving the majority of infants unprotected from RSV [13–15]. Therefore, the development of new RSV prevention strategies is becoming a global health priority [13, 16]. Most recently, two new products for the prevention of RSV disease in infants have been authorized [3]. Nirsevimab is a long-acting monoclonal antibody approved on 31 October 2022 in the European Union for the prevention of lower respiratory tract disease caused by RSV in newborns and infants during their first RSV season [17]. Instead, RSV vaccine (bivalent, recombinant) is a vaccine for use in pregnancy for the prevention of lower respiratory tract disease in infants from birth through 6 months of age; the vaccine was authorized in the European Union on 23 August 2023 [18].

Planning and adopting such new prevention strategies (i.e., vaccination or long-acting monoclonal antibodies), require accurately updated burden estimates and careful identification of those children at higher risk of RSV-associated hospitalization. Furthermore, these estimates may be useful to evaluate the post-marketing impact of new products on the prevention of RSV. In this study, we aimed to describe the clinical and epidemiological characteristics of RSV-associated hospitalizations among children in pediatrics or NICU of the Policlinico Foggia Hospital, Apulia region, Southern Italy.

## Methods

### Study design

The present retrospective observational study included children with a laboratory-confirmed RSV infection hospitalized in pediatrics or NICU of the Policlinico Foggia Hospital.

Laboratory-confirmed RSV-associated hospitalizations were identified from the RSV Surveillance Register created at the Microbiology and Virology Section of the Policlinico Foggia Hospital since 2011. RSV infection was ascertained through the use of real-time PCR (Allplex™ Respiratory Panel 4). We included in the study all cases from 2011 to 2023. Using cases from the RSV Surveillance Register, we described the seasonal trend of RSV-associated hospitalization.

### Clinical characteristics of RSV-associated hospitalizations, 2011–2020

RSV Surveillance Register records from 2011 to 2020 were matched with the corresponding hospital admissions in the Hospital Discharge Registry (HDR) of the pediatrics or NICU. A personal identification (ID) number and hospital admission date were used as linkage keys for data matching. Table 1 reports International Classification of Disease, Ninth/Tenth Revision, Clinical Modification (ICD-9CM) codes used to identify (in primary or secondary diagnoses) the clinical categorization and the severity measures.

Furthermore, the RSV database 2011–2020 was linked with the Apulian HDR from 2003 (first year of birth of children included in the study) to 2020 to collect information on chronic comorbid conditions and history of prematurity. A personal ID number was used as a linkage key. Children with chronic illness were classified using the pediatric Complex Chronic Conditions (CCCs) classification system (v2) [19, 20], that represents the “gold standard” for classifying children with comorbidities. Such method uses the diagnosis codes of the ICD-9-CM or ICD-10-CM to create CCC categories that have a high likelihood of meeting the CCC definition: neuromuscular, cardiovascular, congenital, respiratory, gastrointestinal, renal, metabolic, hematological, cancer and a category of perinatal conditions (premature and neonatal) [19, 20]. Children with any of these nine conditions are considered to have a CCC [19, 20]. The presence of CCCs was considered only if the hospital admission happened concomitantly or prior to the RSV-associated hospitalization.

The quality of matching was assessed by estimating linkage error rates, in order to understand the mechanisms by which these errors may have affected and biased the results.

### Clinical and economic burden of RSV-associated hospitalizations

The burden of RSV-associated hospitalizations on the overall hospitalizations due to acute respiratory infections (ARIs) admitted in Policlinico Foggia Hospital during the study period 2011–2020 was estimated by calculating the proportion of RSV-associated hospitalizations in pediatrics and NICU on the total hospitalizations for ARI in the same units and period. ICD-9-CM codes used to identify the ARIs-associated hospitalizations are reported in Supplementary Table 1. The hospitalizations cost analysis was performed using diagnosis-related group (DRG) reimbursement rates considered by the National Health System. Such rates are a proxy of the actual cost of hospitalizations and they represent the actual expenditure of the healthcare system by the public sector. The cost was reported as mean annual cost per patient, age classes (0–2 months vs. >2 months) and unit.

### Estimate of the potential impact of nirsevimab and RSV vaccine (bivalent, recombinant)

The potential impact of the new immunization programs with nirsevimab or RSV vaccine (bivalent, recombinant) was estimated by calculating the average number of hospitalizations cases that could be prevented if the whole population was immunized. For performing such calculations, the average number of yearly RSV-related hospitalizations in children under 1 year of age between 2011 and 2019 (excluding the first year of the pandemic) were multiplied for the efficacy data of monoclonal antibodies and maternal immunization against RSV hospital admission as reported in the literature (77.3% for nirsevimab [21] and 69.4% for RSV vaccine (bivalent, recombinant) [22]). The potential impact of nirsevimab was also estimated against very severe RSV disease, that included patients with at least one of the following conditions: NICU admission, mechanical ventilation, respiratory therapy, extracorporeal membrane oxygenation [ECMO], deaths (estimated efficacy: 86% [21]).

### Ethics statement

All data included in this study were collected during routine clinical practice, and evaluated retrospectively and anonymously. All procedures performed were in accordance with the ethical standards and the Declaration of Helsinki and within the Italian law. Informed consent was waived because all data were de-identified.

### Statistical analysis

Descriptive statistics were performed. Categorical variables (i.e., sex, age group, year of hospitalization, type specimens, RSV subtypes, admission unit, clinical categorization, severity measures, delivery during the RSV infection season, CCCs, history of prematurity) were expressed as counts and percentages in each category. Age classification was performed according to Rha et al. [15], to highlight the proportion of cases under 1 year of age and especially 0–2 months of age. Frequencies were expressed as percentages with 95% confidence interval (CI) calculated using the Clopper − Pearson method. Continuous variables (i.e., age, length of hospital stay) were defined as mean (Standard Deviation [SD]) and median with interquartile range (IQR).

The association between qualitative variables was assessed by calculating the chi-squared test (χ2) and odds ratios (ORs) with 95%CIs. Differences in continuous variables were tested with Student’s t-test for normally distributed variables, or with the Mann-Whitney U-test when variables showed a non-normal distribution. Normality of data was tested using the Kolmogorov-Smirnov test.

When the univariate analysis revealed significant differences in some variables, these were included in a multivariate logistic regression model to evaluate whether demographic (i.e., sex, age) or clinical (i.e., RSV subtypes, clinical categorization, delivery during the RSV infection season, CCSs and history of prematurity) characteristics were independently associated with the severity of RSV-derived hospitalizations. In addition, a further multivariate model was used to assess the association between demographic/clinical characteristics and RSV subtypes. The level of statistical significance was set at *p* < 0.05. All analyses were performed using STATA/SE 15.0.

## Results

### RSV-associated hospitalizations characteristics, 2011–2023

Between January 2011 and December 2023, 1,816 inpatients with a laboratory-confirmed diagnosis of RSV infection were recorded in the RSV Surveillance Registry of Policlinico Foggia Hospital. A total of 1,005 patients were admitted to pediatrics or NICU units and included in the study. The other 811 confirmed cases were admitted to different units. Of the cases included in the study, 564 (56.1%) were male and 736 (73.2%) were aged < 1 year. RSV-A was the prevalent RSV subtype detected in 538 patients (53.5%).

### Seasonal trend of RSV-associated hospitalization

**Fig. 1:**
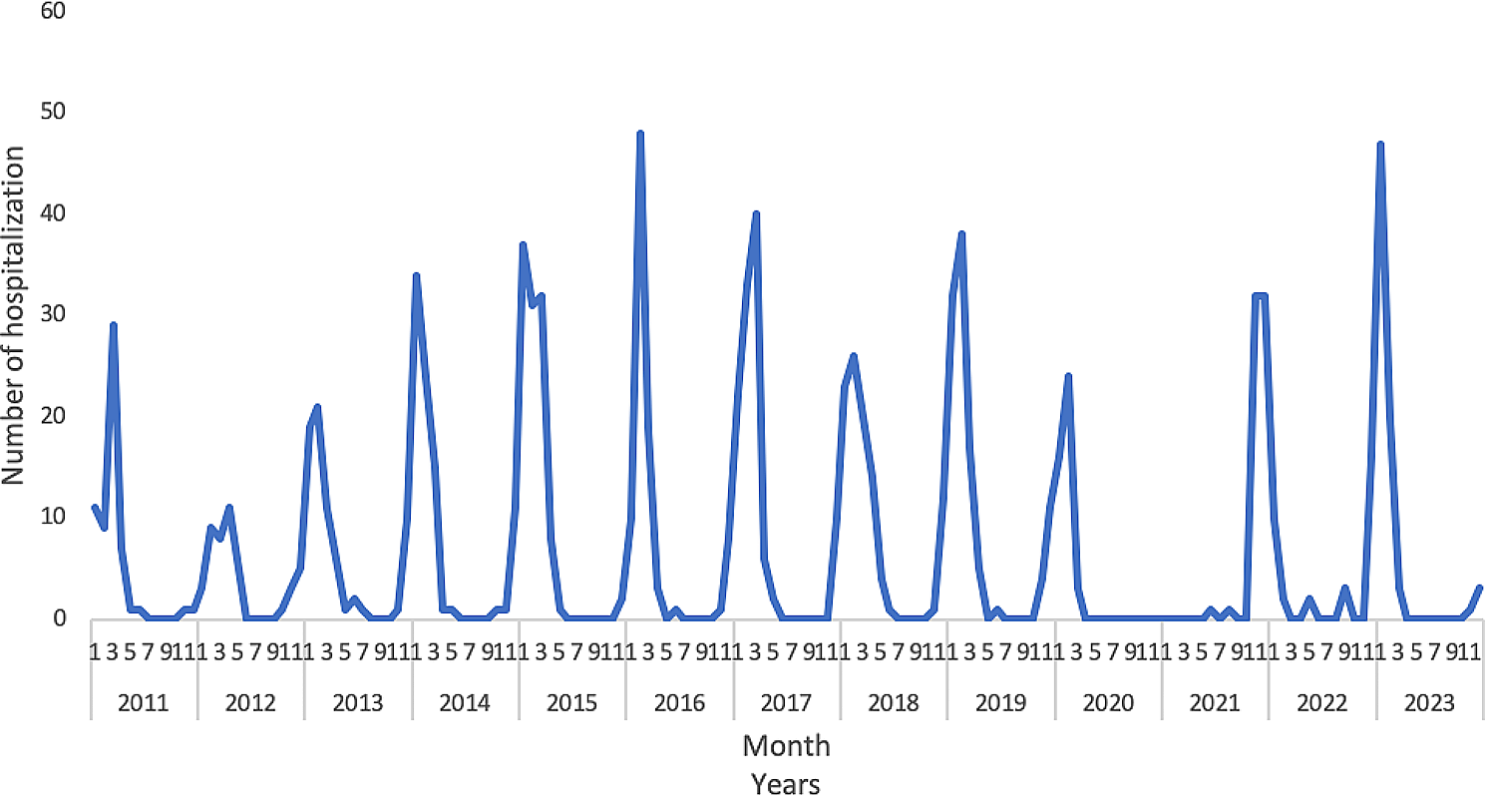
Seasonal trend of RSV-associated hospitalization. Policlinico-Foggia-Hospital, Apulia region, Italy, 2011 − 2023

**Fig. 2:**
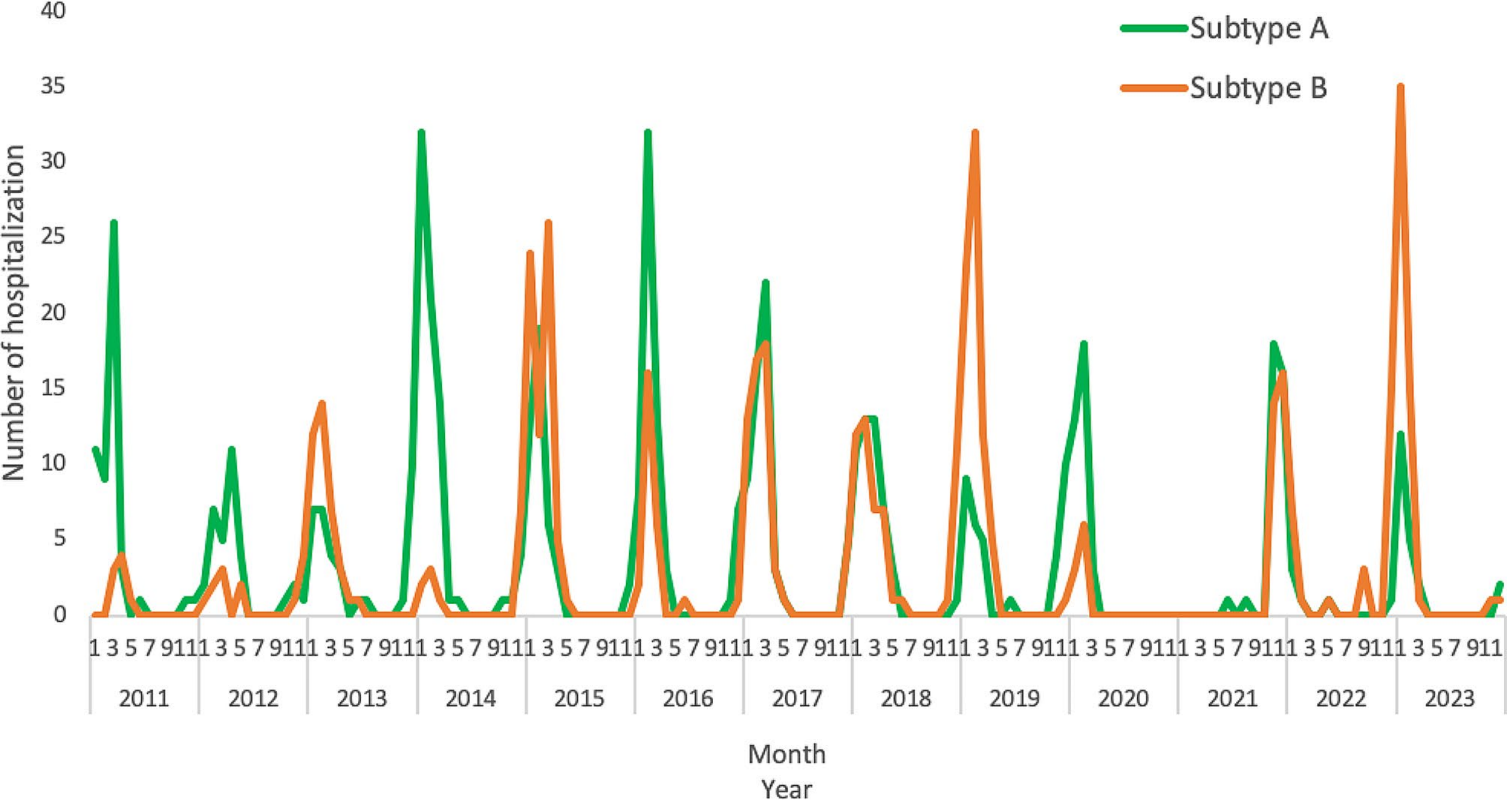
Seasonal trend of RSV-associated hospitalization per subtypes. Policlinico-Foggia-Hospital, Apulia region, Italy, 2011 − 2023

Figure 1 shows the seasonal trend of RSV hospitalization. The epidemic period of RSV starts in November, has a peak between January and February, and ends in April; 867 cases (86.3%) were recorded between December and March. During the COVID-19 pandemic a reduction in hospitalizations due to RSV was observed. Figure 2 shows that the subtype prevalence varies according to the epidemic season. In almost all seasons, the prevalent subtype was RSV-A, except for 2013, 2015, 2019, 2022 and 2023 where the prevalent subtype was RSV-B.

### Clinical characteristics of RSV-associated hospitalizations, 2011–2020

A total of 832 RSV cases recorded in the RSV Surveillance Registry between January 2011 and December 2020 were matched with the corresponding hospital admissions in the HDR of the pediatrics or NICU. No error was reported in the matching procedure.

Of these 832 RSV cases, 470 (56.5%) were male and 630 (75.2%) were aged < 1 year (49.6% 0–2 months of age). Regarding diagnosis, 63.3% and 18.3% of hospital discharge records were codified as acute bronchiolitis and pneumonia due to RSV, respectively. In particular, bronchiolitis represented 76% (481/630) of the total clinical diagnosis among the RSV-positive cases up to one year of age. The median length of hospital stay was 5 days (IQR 3–7 days); 179 patients (21.5%) were admitted to the NICU, 35 (4.2%) required mechanical ventilation, 99 (11.9%) received other respiratory therapy, and one death occurred. Chronic comorbid conditions and a history of prematurity were documented in 125 (15%) and 70 (8.4%) children, respectively. The demographic and clinical characteristics of patients admitted between 2011 and 2020 are summarized in Table 1.

**Table 1:** Demographics and clinical characteristics of RSV-hospitalized patients 2011–2020. Policlinico Foggia, Apulia region, Italy

Characteristics	Number* (*N* = 832)	(%)	95% CI
Sex			
Male	470	56.5	53.0–59.9
Female	362	43.5	40.1–46.9
**Age, months**			
*Median age (IQR)*	3 (1–11)		
**Age group (months)**			
0–2	413	49.6	46.4–53.3
3–5	121	14.5	12.2–17.1
6–11	96	11.5	9.6–14.0
12–23	96	11.5	9.0–13.4
24–59	97	11.7	9.0–14.2
> 59	9	1.1	0.5–2.0
**Hospitalization year**			
2011	60	7.2	5.6–9.2
2012	46	5.5	4.2–7.3
2013	69	8.3	6.5–10.3
2014	89	10.7	8.7–12.9
2015	113	13.6	11.4–16.0
2016	90	10.8	8.9–13.1
2017	113	13.6	11.4–16.1
2018	98	11.8	9.7–14.1
2019	111	13.3	11.1–15.8
2020	43	5.2	3.8–6.9
**Type specimens**			
Nasopharyngeal swab	811	97.5	96.0–98.3
Bronchoalveolar lavage fluid	10	1.2	0.6–2.2
Tracheal wash	11	1.3	0.7–2.3
**RSV subtypes**			
A	474	57.0	53.6–60.3
B	358	43.0	39.6–46.4
**Admission unit**			
Pediatric	653	78.5	74.3–81.2
Neonatal intensive care	179	21.5	18.8–24.6
**Clinical categorization**			
Bronchiolitis	527	63.3	59.9–66.6
Pneumonia	152	18.3	15.7–21.1
Acute upper respiratory infections due to RSV	67	8.1	6.3–10.1
Unspecified respiratory disease due to RSV	37	4.4	3.11–6.1
Unspecified respiratory disease	49	5.9	4.39–7.71
**LOS**			
*Median (IQR)*	5 (3–7)		
*Frequency per days*			
0–1 days	20	2.4	1.5–3.7
2 days	86	10.3	8.3–12.6
3–4 days	260	31.2	28.1–34.5
≥ 5 days	466	56.0	40.7–47.6
Very severe RSV disease*	208	25.0	22.1–28.1
2011	14	6.7	3.7–11.0
2012	13	6.3	3.4–10.4
2013	17	8.2	4.8–12.7
2014	18	8.7	5.2–13.3
2015	16	7.7	4.5–12.2
2016	15	7.2	4.1–11.6
2017	35	16.8	12.0-22.6
2018	32	15.4	10.8–21.0
2019	35	16.8	12.0-22.6
2020	13	6.3	3.4–10.4
**Severity measures**			
NICU admission	179	21.5	18.8–24.6
Mechanical ventilation	35	4.2	2.9–5.8
Respiratory therapy	99	11.9	9.8–14.3
ECMO	0	0	-
Deaths	1	0.1	0.003–0.7
**Delivery during the RSV infection season**	532	63.9	60.6–67.2
**Comorbid condition**			
Pediatric CCCs	125	15.0	12.7–17.6
History of prematurity	70	8.4	6.6–10.5

CCCs: complex chronic conditions; CI: confidence interval; ECMO: extracorporeal membrane oxygenation; IQR: interquartile range; LOS: length of hospital stay; NICU: neonatal intensive care unit; RSV: respiratory syncytial virus

*Al least one condition: neonatal intensive care unit admission, mechanical ventilation, respiratory therapy, extracorporeal membrane oxygenation, death

Multivariate logistic regression revealed that younger age (≤ 2 months) (OR: 14.8, 95% CI: 8.30– 26.31, *p* = 0.000), higher length of hospital stay (OR: 1.01, 95% CI: 1.0–1.02, *p* = 0.030) and history of prematurity (OR: 4.4, 95% CI: 1.57–12.11, *p* = 0.005) were significantly related to very severe RSV disease (Table 2a). On the contrary, no statistically significant association of hospitalization severity with delivery during the RSV infection season (OR: 1.4, 95% CI: 0.82–2.53, *p* = 0.192) nor pediatric CCCs (OR: 1.0; 95% CI: 0.42–2.44, *p* = 0.957) was found.

Furthermore, the data presented in Table 2b show no statistically significant association between any of the demographic/clinical characteristics and RSV subtype.

### Clinical and economic burden of RSV-associated hospitalizations

Overall, a total of 19,467 hospitalizations occurred in the Policlinico Foggia Hospital between 2011 and 2020 in the pediatrics and NICU. The admissions for ARIs in these units were 3,972 (20.4%), of which 832 (20.9%) were associated with RSV. Among children younger than 1 year, the number of hospitalizations for ARI was 1,287 (32.4%), of which 630 (48.9%) were caused by RSV.

The overall cost of RSV-related hospitalizations in the study period (2011–2020) corresponded to 2,525,966.00 euros. The mean cost of a single RSV-associated hospitalization corresponded to 3,036 euros/year (95% CI: 2,643–3,428). The mean cost of RSV-associated admissions in NICU (7,732 euros/year,95% CI: 6,110–9354) was significantly higher than that of pediatrics admissions (1,748 euros/year, 95% CI: 1,650–1,846; *p* = 0.0000). The average cost of hospitalization among children aged 0–2 months (4,225 euros/year,95% CI: 3,474–4,977) was higher than that for older children (1,863 euros/year, 95% CI: 1,675–2,050; *p* = 0.0000).

### Estimate of the potential impact of nirsevimab and RSV vaccine (bivalent, recombinant) in the Foggia context

Excluding the first year of the Covid-19 pandemic, between 2011 and 2019, the average number of RSV-related hospitalizations among patients aged under 1 year admitted in the children’s wards of the Policlinico Foggia Hospital was 66.5. Based on literature data, nirsevimab has an efficacy of 77.3% against RSV hospital admission [21]. Therefore, 51.4 hospitalizations/year (i.e., 20% of ARI-related hospitalization) could have been avoided by immunization with nirsevimab. On another note, an 86% efficacy of nirsevimab against very severe RSV disease is reported in the literature [21]. Among children younger than 1 year, an average of 21 hospital admissions for very severe RSV per year were recorded. Thus, immunization with the monoclonal antibody could have prevented 18.1 severe RSV hospitalization per year. Similarly, a vaccine efficacy of 69.4% of preventing severe lower respiratory tract illness within 180 days after birth was demonstrated for RSV vaccine (bivalent, recombinant) in pregnancy [22]. Consequently, 46.1 hospitalizations (corresponding to 17% ARI-related hospitalization) per year could have been avoided by immunizing the whole pregnancy population resident in Foggia before the start of the RSV season.

**Table 2a:** Univariate/multivariate analysis assessing association between demographic/clinical characteristics and severe RSV-disease. Policlinico-Foggia-Hospital, Apulia region, Italy, 2011 − 2020

	Very severe RSV disease*		Univariate Analysis			Multivariate Analysis		
	Yes (*N* = 208)	No (*N* = 624)						
Characteristics	n (%)/mean (SD)	n (%)/mean (SD)	OR	95% CI	p value	OR	95% CI	p value
**Sex, n (%)**								
Male	119 (57.2)	351 (56.2)			Ref.			
Female	89 (42.8)	273 (43.8)	1.03	0.75–1.45	0.8086			
**Age group (months), mean (SD)**	1.8 (8.7)	10.9 (14.7)			0.0000	14.8	8.30–26.31	0.000**
**LOS (days), mean (SD)**	10.02 (17.47)	5.59 (14.87)			0.0002	1.01	1.0–1.02	0.030***
**RSV subtype, n (%)**								
A	116 (55.8)	358 (57.4)	-	-	Ref.			
B	92 (44.2)	266 (42.6)	0.94	0.67–1.30	0.6860			
**Delivery during the RSV infection season**	182 (87.5)	350 (56.1)	5.5	3.49–8.86	0.0000	1.4	0.82–2.53	0.192
**Comorbid condition**								
Pediatric CCCs	51 (24.5)	74 (11.9)	2.4	1.58–3.65	0.0000	1.0	0.42–2.44	0.957
History of prematurity	41 (19.7)	29 (4.6)	5.0	2.9–8.6	0.0000	4.4	1.57–12.11	0.005

CCCs: complex chronic conditions; CI: confidence interval; LOS: length of stay; RSV: respiratory syncytial virus; SD: standard deviation

*Al least one condition: neonatal intensive care unit admission, mechanical ventilation, respiratory therapy, extracorporeal membrane oxygenation, death

**Age 0–2 months vs. >2 months

***LOS included in the model as days

**Table 2b:** Univariate/multivariate analysis assessing association between demographic/clinical characteristics and RSV-subtype. Policlinico-Foggia-Hospital, Apulia region, Italy, 2011 − 2020

	RSV subtype		Univariate Analysis			Multivariate Analysis		
	A (*N* = 474)	B (*N* = 358)						
Characteristics	n (%)/mean (SD)	n (%)/mean (SD)	OR	95% CI	p value	OR	95% CI	p value
**Sex, n (%)**								
Male	215 (45.4)	147 (41.1)			Ref.			
Female	259 (54.6)	211 (58.9)	0.84	0.62–1.11	0.2158			
**Age group (months), mean (SD)**	7.6 ± 12.5	10.1 ± 15.7	-	-	0.0050	1.02	0.71–1.45	0.926*
**LOS (days), mean (SD)**	6.22 ± 10.23	7.34 ± 20.78	-	-	0.1564			
**Severity measures, n (%)**	116 (55.8)	358 (57.4)	0.94	0.67–1.30	0.6860			
**Delivery during the RSV infection season**	301 (63.5)	231 (64.5)	0.95	0.71–1.28	0.7609			
**Comorbid condition**								
Pediatric CCCs	75 (15.8)	50 (14.0)	1.15	0.77–1.74	0.4581			
History of prematurity	42 (8.9)	28 (7.8)	1.14	0.67–1.96	0.5928			

CCCs: complex chronic conditions; CI: confidence interval; LOS: length of stay; RSV: respiratory syncytial virus; SD: standard deviation

*Age 0–2 months vs. >2 months

## Discussion

This study analyzed the characteristics of RSV-infected infants and young children hospitalized at the Policlinico Foggia Hospital between 2011 and 2023.

In terms of virus seasonality, our study found an increased RSV-associated hospitalization between December and March with a peak in January/February. The observed seasonal trend is in line with RSV seasonality reported in the literature in Italy and temperate Northern Hemisphere [9, 10, 23]. Indeed, it is well known that RSV circulation is highly dependent on geographic location. Possible reasons behind RSV seasonal trend include enhanced exposure due to increased indoor crowding during the cold months, higher RSV stability in fomites at lower temperature and low absolute humidity/UVB irradiation causing increased RSV infection risk [24]. The prevalence of a RSV-subtype over the other varies based on the epidemic season; according to Pierangeli et al. RSV-A dominated in 2017–2018, 2019–2020 and 2021–2022 epidemic seasons [25]. Our study found a higher circulation frequency of RSV-A than RSV-B (53.5% vs. 46.5%) and no statistically significant association between RSV subtype and demographics and clinical characteristics. This finding was consistent with other studies that did not report significant difference in the distribution of different subtypes according to sex, age, length of hospital stays or any comorbidity/prematurity [26–31]. The association between the RSV subtype and clinical severity has been the subject of research for an extended period. As in our study, inconsistent associations between RSV subtype and clinical severity have been reported in the literature, although the majority of previous studies report a higher risk of intensive care treatment or more severe clinical outcome in patients infected with RSV subtype A [25, 28, 31, 32]. Several factors may contribute to explain the association between RSV subtype and clinical severity. The presence of coinfections may complicate the clinical outcome and make it difficult to attribute specific symptoms or severity solely to the RSV subtype [28]. In addition, individual variations in the host immune response, including the presence of maternal antibodies, may influence the severity of RSV infection. The ability of the RSV to mutate and adapt may contribute to the variability in disease severity, making it difficult to establish a direct relationship between subtype and clinical impact [33–36].

Additionally, our study found a decrease of RSV-related admissions during the COVID-19 pandemic from 2020 to 2021. Our result agrees with the literature that shows a reduction in the incidence of RSV infections/hospitalization during the pandemic followed by an increase in the post-pandemic period [25, 37–40]. Indeed, the restrictive measures adopted during the COVID-19 pandemic have modified the epidemiology/seasonality of other pathogens including RSV and created an immune debt [41].

In the period 2011–2020, the majority of the hospitalized patients in our study (50%) were under 2 months of age in line with other international published studies that have also found the highest age-specific hospitalization number in children aged 0–2 months [5, 29, 42, 43]. The increased incidence of RSV infection in the first months of life is believed to be related to a weak maternally derived immunity [44]. Such age-specific hospitalization trend highlights the need for immunization programs targeted at the first months of life. A slight difference in the frequency of hospitalizations was noted between male and female subjects (56.5% vs. 43.5%). A higher vulnerability for the male sex is indeed reported in the literature due to immuno-modulatory effects of male sex hormones during neonatal age [45].

The analysis of clinical data showed that bronchiolitis was the most frequent RSV-related admission diagnosis, confirming the well-known notion that bronchiolitis is the most common severe clinical manifestation of RSV infection, especially in children under the age of two years [46, 47]. In particular, we found that bronchiolitis was the most frequent clinical diagnosis among RSV-positive patients under 1 year of age (76%), in line with the result (86%) of a recent Italian multicenter study by Pierangeli et al. [48].

Only 10.5% of RSV-hospitalized cases were born preterm. Our results are consistent with other recent epidemiological studies showing that most children with RSV infection requiring a medical visit at a pediatric practice or hospitalization were healthy and born at term [9, 10, 15, 42, 49, 50]. However, our analysis found a correlation between the severity of hospitalization measures and a history of prematurity. A study aimed to identify the risk factors of severe RSV disease in children born before 34 weeks of gestation: younger corrected age was identified as the main risk factor for severe disease in preterm children [51]. Another recent study showed that infants born prematurely had the highest risk of NICU admission [50]. The pathophysiology of bronchiolitis may help explain such findings: the impact of virus-induced mucus accumulation and bronchiole obstruction is bigger in babies who already present a low diameter of the bronchioles. Furthermore, prematurity is associated with a compromised passive immunity [52].

In our study, RSV caused around 50% of ARI-related hospitalizations among children under 1 year of age, confirming the elevated burden of RSV infection in the first year of life. Moreover, our findings confirmed that a very severe RSV disease is associated with a prolonged length of hospital stay (10 days vs. 5.6 days), as already reported in the literature [53]. The hospitalization cost for acute bronchiolitis in Europe is about 2,000 euros per patient in pediatric wards and 8,000 euros per patient in pediatric intensive care units. In accordance with the European figure, our study shows a significantly higher hospitalization cost for NICU patients and children aged ≤ 2 months [8, 54]. Therefore, the adoption of prevention strategies aimed at protecting children under 1 year of age could have a significant impact in reducing the incidence of hospitalization, the length of hospital stay and, hence, associated healthcare costs. A recent publication illustrates how the benefits of RSV immunization go beyond the prevention of acute respiratory illness in the first year of life. Increasing evidence shows that the prevention of RSV infection in infants under 1 year of age could also prevent secondary pneumonia caused by other pathogens and reduce hospitalization resulting from other respiratory diseases in later childhood. Additional secondary benefits include a reduction of overall infant mortality, a lower healthcare burden due to respiratory diseases, prevention of inappropriate antibiotic use, and improved long-term lung health [55]. According to the efficacy data of immunization strategies, we could have prevented about 70% of RSV hospital admission and more than 85% of very severe RSV disease. However, despite the European Medicines Agency (EMA) approval, the evident need for immunization and strong scientific evidence on clinical and public health benefits of RSV prevention, worrisome disparities have emerged in the immunization programs across Europe with the risk of jeopardizing the achievement of a population immunity. Italy is among those countries where RSV immunization is suffering a delay due to additional local regulatory steps and complicated bureaucracy [56]. A first real-world evidence of nirsevimab immunization, within a context of moderate to high coverage (84%) among newborns, has shown in 2023 a decrease of 38% in hospitalizations under 5 years of age and 69% in hospitalizations of infants under 6 months old compared to 2022 [57].

The results of our study should be interpreted considering some limitations. Firstly, for the last three years, we included in the study only epidemiological data collected from the RSV Surveillance of the Microbiology and Virology Section of Policlinico Foggia, as the Hospital Discharge Registry for the years 2021–2023 was not available. Therefore, for cases discharged in these years, we were unable to evaluate clinical information, the proportion of overall hospitalizations for ARIs associated with RSV infection, hospitalization costs, and the anticipated impact of immunization. Secondly, patient clinical categorization is exclusively based on the information included in the hospital discharge form precluding accurate collection of clinical data. So, as no outpatient data are available, comorbidity evaluation is only available for those patients that were previously hospitalized in the Apulia region. An additional limitation of our study consists in the lack of an identified denominator to calculate hospitalization rates. Another limitation of our study is the lack of information on whether hospitalised infants had previously received palivizumab. As a result, we were unable to assess the effect of this preventive measure in our case sample, including assessing on the potential impact of immunization with RSV vaccine and passive immunization by administration of nirsevimab. It is important to note that our assessment of the potential impact of the use of nirsevimab and RSV vaccines was limited to the data obtained from the Policlinico Foggia. Therefore, the available evidence may not be sufficient to estimate the impact in the whole District of Foggia, considering that the impact depends on the level of immunization and vaccination coverage achieved in the population.

## Conclusions

Our results confirm that RSV is a main leading cause of hospitalization in infants, with a peak between January and February. Most of cases occurred among 0 to 2 months of age. Although preterm children had a higher risk of severe disease, most hospitalization occurred in children born at term. These findings enable a baseline characterization of RSV-associated hospitalizations among Italian children, providing epidemiological, clinical, and economic burden estimates. While awaiting the adoption of new recommendations on passive immunization and maternal vaccination, it is crucial that healthcare workers remain committed to implementing general public health measures and ensuring appropriate case management to control RSV seasonal epidemic picks. Future studies using a laboratory-based approach and enhanced routine RSV surveillance are warranted to optimize public health response and support policymakers in implementing future immunization programs.

### Electronic supplementary material

Below is the link to the electronic supplementary material.

Supplementary Material 1

## Data Availability

All relevant data are within the manuscript/The data used to support the findings of this study are available from the corresponding author upon request.

## Abbreviations

RSV: Respiratory syncytial virus
NICU: Neonatal intensive care unit
HDR: Hospital Discharge Registry
ICD-9/10-CM: International Classification of Disease, Ninth/Tenth Revision, Clinical Modification
CCSs: Complex Chronic Conditions
ARIs: Acute respiratory infections
DRG: Diagnosis-related group
ECMO: Extracorporeal membrane oxygenation
CI: Confidence interval
SD: Standard Deviation
IQR: Interquartile range
ORs: Odds ratios
LOS: Length of stay
EMA: European Medicines Agency
