# Supplementary material for: Respiratory syncytial virus-associated hospitalizations among children: an Italian retrospective observational study
Source: Ital J Pediatr. 2024 Mar 7;50:45. doi: 10.1186/s13052-024-01617-w (PMC10921699; doi:10.1186/s13052-024-01617-w)
Supplement: Supplementary file 1 — Supplementary Material 1 [file 13052_2024_1617_MOESM1_ESM.docx]

Supplementary Table 1

| **Description** | **Type** | **Code** |
| --- | --- | --- |
| ***Clinical categorization*** |  |  |
| Acute bronchiolitis due to RSV | ICD-9-CM | 079.6 and/or 466.11; 079.6 and 466.XX |
| Pneumonia due to RSV | ICD-9-CM | 079.6 and/or 480.1; 079.6 and 480.XX-486.XX |
| Acute upper respiratory infections due to RSV | ICD-9-CM | 079.6 and 460.XX-465.XX |
| Unspecified respiratory disease due to RSV | ICD-9-CM | 079.6 without other codes |
| Unspecified respiratory disease | ICD-9-CM | 460.XX-466.XX; 480.XX-487.XX (excluded 079.6, 466.11, 480.1); 769.XX; 786.XX |
| **Severity measures** |  |  |
| Mechanical ventilation | ICD-9-CM | 96.7X |
| Respiratory therapy | ICD-9-CM | 93.9X |
| ECMO | ICD-9-CM | 39.65 |
| **Acute respiratory infections** | ICD-9-CM | 460.XX-466.XX; 480.XX-487.XX; |

ECMO: extracorporeal membrane oxygenation; ICD-9-CM: international classification of diseases, Ninth Revision, Clinical Modification; RSV: respiratory syncytial virus
